# Platelet rich fibrin three-layer reconstruction of the sellar floor after endoscopic endonasal transsphenoidal approach: technical note and initial experience

**DOI:** 10.3389/fsurg.2024.1500158

**Published:** 2024-12-03

**Authors:** Mukesch Johannes Shah, Katerina Argiti, Julia M. Nakagawa, Angeliki Stathi, Emilia Schönen, Daniel Strahnen, Kevin Joseph, Jakob Straehle, Nicolas Neidert, Jürgen Beck, Ioannis Vasilikos

**Affiliations:** ^1^ Department of Neurosurgery, Faculty of Medicine, Medical Center, University of Freiburg, Freiburg, Germany; ^2^ Medical Faculty, University of Freiburg, Freiburg, Germany; ^3^ Laboratory of Experimental Brain & Spine Surgery (LENS), Faculty of Medicine, Medical Center, University of Freiburg, Freiburg, Germany; ^4^ Berta-Ottenstein Programme, Faculty of Medicine, University of Freiburg, Freiburg, Germany

**Keywords:** platelet rich fibrin, endoscopic endonasal transsphenoidal, sellar floor reconstruction, cerebrospinal fluid leak, pituitary adenoma, autologous biomaterial

## Abstract

**Background:**

Cerebrospinal fluid (CSF) leakage frequently complicates endoscopic endonasal transsphenoidal pituitary resections, despite the use of lumbar drains, nasoseptal flaps, or commercial dura sealants. Managing this complication often requires revision surgery and increases the risk of infection. Platelet-rich fibrin (PRF), an affordable autologous biomaterial derived from the patient's blood through short, angulated centrifugation, contains growth factors and leukocytes embedded in a fibrin matrix. PRF exhibits regenerative properties in various surgical disciplines. This study assesses a three-layer sellar reconstruction method employing solid membranous (s-PRF) and high-viscosity injectable (i-PRF) forms of PRF.

**Materials and methods:**

We present our initial experience on a series of 22 patients with pituitary macroadenomas. For all patients, an endoscopic transnasal transsphenoidal approach was selected. Following the resection of the pathology, sellar reconstruction was accomplished using a three-layer orthobiologic technique. A membranous s-PRF was utilized as an inlay inside the opened sellar floor, followed by a layer of injectable i-PRF finally covered with another s-PRF membrane over the top to the sellar corridor.

**Results:**

In all cases the implementation of the proposed three-layer PRF reconstruction strategy was feasible and safe. During the 12-month follow-up period there were no adverse effects reported associated with the PRF application. 77% (17/22) of the patients demonstrated intraoperatively a cerebrospinal fluid (CSF) leak (Esposito Grade 1–3). In total, the proposed PRF reconstruction effectively prevented postoperative CSF leaks in 95% of the patients and in 94% of those with an Esposito Grade 1–3. One of the two patients with intraoperative Esposito Grade 3 developed a CSF leak on the first postoperative day, which was successfully managed with a lumbar drain for 5 days.

**Conclusion:**

Sellar reconstruction after endoscopic endonasal transsphenoidal resection of pituitary adenomas with PRF is feasible and safe. The three layer PRF augmentation is a novel technique to prevent CSF-leakage.

## Introduction

The current standard of care for the reconstruction of the sellar floor following endonasal endoscopic transsphenoidal (EET) resection of pathologies is predicated on a combination of synthetic or autologous biomaterials, with the objective of achieving watertight closure and preventing cerebrospinal fluid (CSF) leaks. Despite the introduction of novel protocols, the incidence of CSF leaks subsequent to EET procedures ranges from 2%–30%, potentially resulting in severe postoperative complications, necessitating reoperations, and leading to extended periods of hospitalization and rehabilitation (1–3).

Over time, the reconstruction strategies for sellar floor repair following EET surgery have undergone significant evolution. The current options encompass fat grafts, mucosal or vascularized nasoseptal flaps, and synthetic dural substitutes (4–7). Nasoseptal flaps have been frequently employed in larger defects and have demonstrated a reduction in the incidence of postoperative cerebrospinal fluid (CSF) leaks (0%–2.9%); however, they have been associated with significant donor site morbidity, nasal crusting, and discomfort (8). The utilization of fat grafts has been also shown to be effective in reducing the postoperative CSF leaks rates but requires harvesting of the graft usually from the periumbilical area (9, 10). Conversely, recent studies have demonstrated the potential of utilizing autologous platelet-rich fibrin for the reconstruction of the sellar floor, exhibiting excellent safety and efficacy profiles (11).

**Figure 1:**
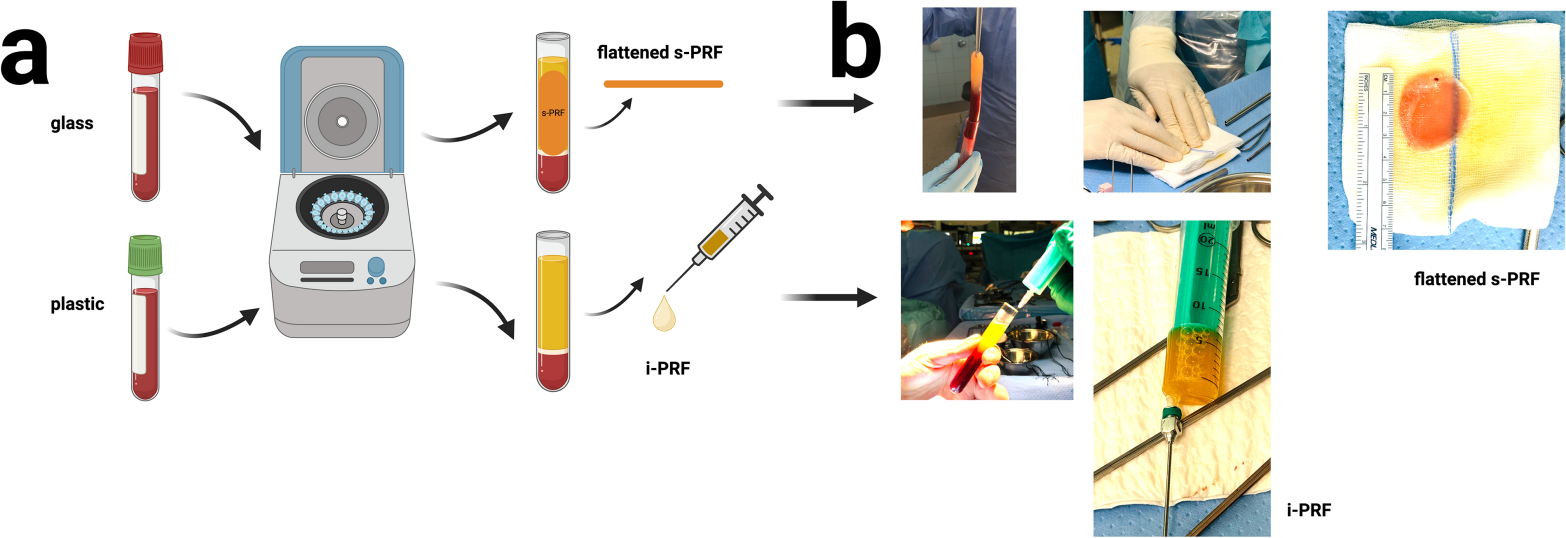
**(a)** schematic representation of the preparation protocol for solid and injectable autologous platelet-rich fibrin, **(b)** intraoperative visualization of the preparation procedure.

Autologous platelet fibrin is readily obtained through a low-speed angulated centrifugation process of the patient's own blood without chemical additives (12, 13). It contains numerous autologous platelet-derived growth factors and chemokines from the patient's own leukocytes, and it has been demonstrated to accelerate healing in various medical disciplines, including plastic, maxillofacial, and orthopedic surgery (13–17). PRF can be prepared in two distinct textures, either fluid or solid, according to the updated protocol of Choukroun and Ghanaati in 2018, following a single cycle of low-speed centrifugation. The solid-PRF (s-PRF, Figure 1: glass vial) comprises an elastic three-dimensional fibrin matrix. It can be mechanically manipulated to achieve the desired shape for its intended application. The injectable-PRF (i-PRF, Figure 1: plastic vial) consists of a fibrin matrix in an intermediate phase. i-PRF exhibits high viscosity and gradually transitions to a gelatinous state upon removal from its preparation vial (18–22). The encapsulated growth factors in PRF are released gradually over a period of up to 14 days (23), supporting the wound healing process and bone regeneration (24), while the leukocytes present provide localized antimicrobial protection (25).

This technical note presents a novel three-layer PRF reconstruction strategy for the sellar floor deficit after EET surgery utilizing s-PRF and i-PRF and elucidates its potential in preventing cerebrospinal fluid leaks.

## Materials and methods

A series of 22 pituitary macroadenoma resections were included. Endonasal endoscopic transsphenoidal resection was performed in all surgeries as indicated by the interdisciplinary skull base board of our institution. Patients undergoing treatment with anticoagulant agents or any medication affecting their coagulation were excluded from this study, as these factors might influence the quality and preparation time of PRF. The degree of intraoperative CSF leak was classified by the operating surgeons based on the work of Esposito et al. as grade 0 (no intraoperative CSF leak), grade 1 (weeping leak), grade 2 (moderate CSF leak), and grade 3 (large CSF leak) (26).

## Preparation of solid and injectable PRF biomaterial

For each patient, blood was obtained from the arterial line at the beginning of surgery. We employed 4 vials (glass) of s-PRF and 2 vials (plastic) of i-PRF, each containing 10 ml of blood, which were centrifuged in a DUO centrifuge (an, Process for PRF, Nice, France), adhering to the established protocol of Choukroun and Ghanaati in 2018 (20, 27, 28). Following centrifugation, the s-PRF matrix (obtained from the glass vials) was manually compressed and flattened on the sterile operating field, while the semifluid version of the i-PRF (obtained from the plastic vials, Supplementary Video S2) was aspirated utilizing a syringe. A detailed protocol of PRF preparation for this study is delineated in our previous work (18, 19) (Figure 1). The PRF preparation process took place during surgery and did not affect the overall duration of surgery.

**Figure 2:**
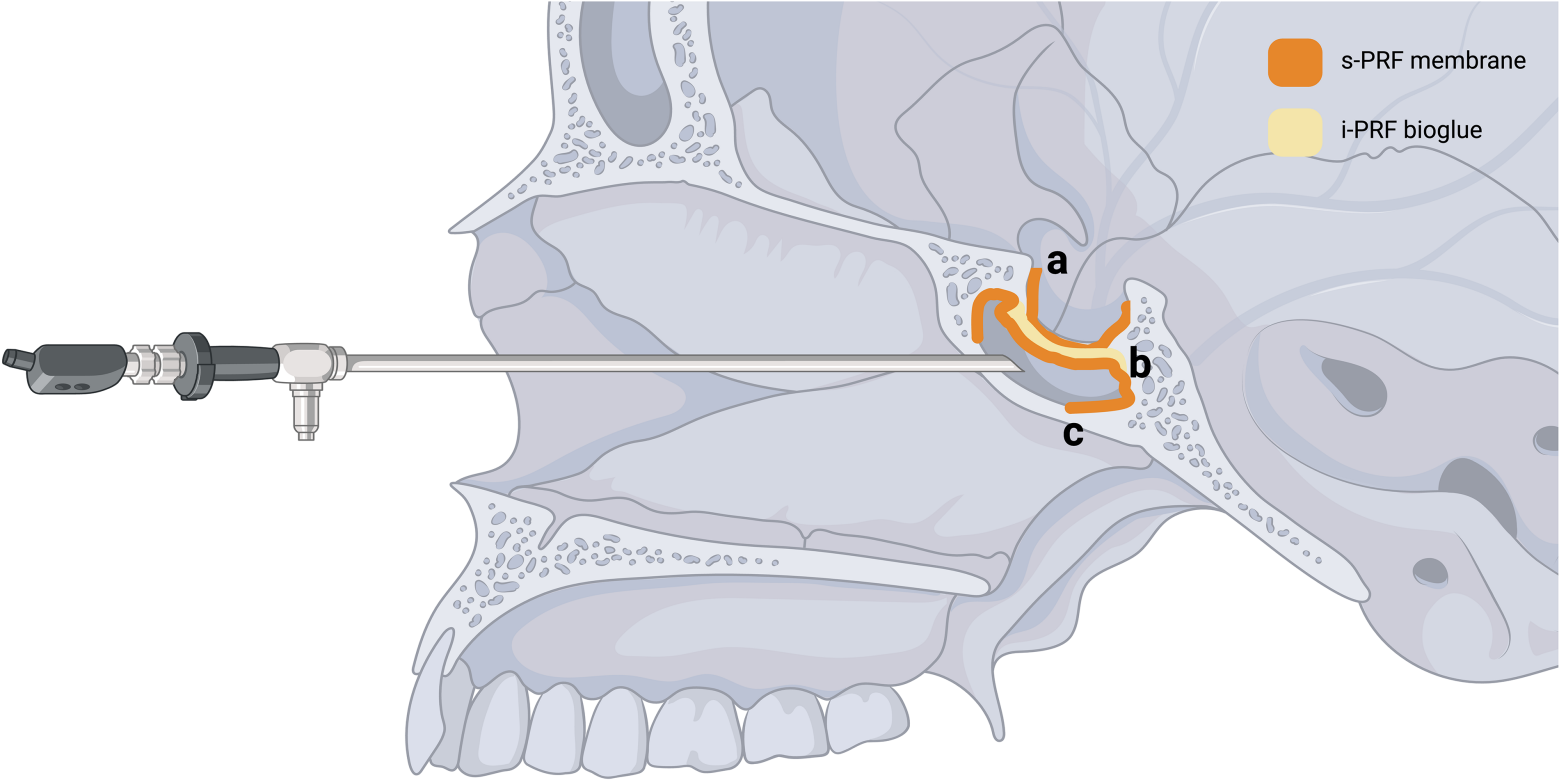
Schematic depiction of the proposed three-layered application of autologous platelet-rich fibrin: **(a)** a flattened solid PRF membrane (s-PRF) is inserted as an inlay at the floor of the sella opening, **(b)** a semi-fluidic autologous injectable PRF glue (i-PRF) is injected on top as a bio adhesive, **(c)** a third layer comprising another s-PRF membrane is applied over the top and lining the sphenoid sinus [created in bioRender. Joseph, K. (2024) BioRender.com/e74u464].

Following the resection of the pathology, the sellar floor was reconstructed using a three-layer PRF approach, as illustrated in Figure 2 and Supplementary Video S1.

Step 1: A flattened s-PRF membrane (Figure 2a) was inserted as an inlay at the floor of the sella.
Step 2: The semi-fluidic autologous i-PRF glue was injected on top as a bioadhesive (Figure 2b).
Step 3: Finally, a third layer comprising another s-PRF membrane was applied over the top and lining the sphenoid sinus, secured in place by a hemostatic gelatin sponge (Gelita-Spon®) (Figure 2c).

Patients were monitored daily for cerebrospinal fluid (CSF) leaks and adverse effects during their postoperative hospitalization period of 5 days, as well as during follow-up examinations in our outpatient clinic at 6 weeks, 3 months, and 1-year post-surgery.

Diagnosis of postoperative CSF leak was established through clinical examination, imaging, and laboratory testing, following a standardized protocol from Constanzo et al. (29).

Diagnosis of intracranial infection was based on clinical examination, CSF analysis, and CSF culture. A negative CSF culture did not preclude infection if clinical signs were present.

Descriptive statistics were employed to present our data in terms of mean (standard deviation), median (interquartile range), or percentage. The collected data are summarized in Table 1.

**Table 1:** Overview of patient characteristics, intraoperative cerebrospinal leak classification based on esposito grade (2) and type of pituitary adenoma (functioning and nonfundctioning).

No. of patients	22
Age (years) (SD)	55.1 ± 13.2
Sex (M:F)	9:12
Intraoperative CSF leak (Esposito grade)	
0	5 (22.7%)
1	1 (4.5%)
2	14 (63.6%)
3	2 (9.09%)
Postoperative CSF leak	1 (4.5%)
Postoperative meningitis	0
Macroadenoma	
Functioning macroadenoma	6 (27.2%)
Nonfunctioning macroadenoma	16 (72.7%)

The study was conducted according to national regulations declaration of Helsinki and its later amendments as well as the Note for guidance on good clinical practise (CPMP/ICH/135/95) (GCP). The study was approved by the local ethics committee (Vote number: 23-1092-S1-retro, 04.04.2023).

## Results

**Figure 3:**
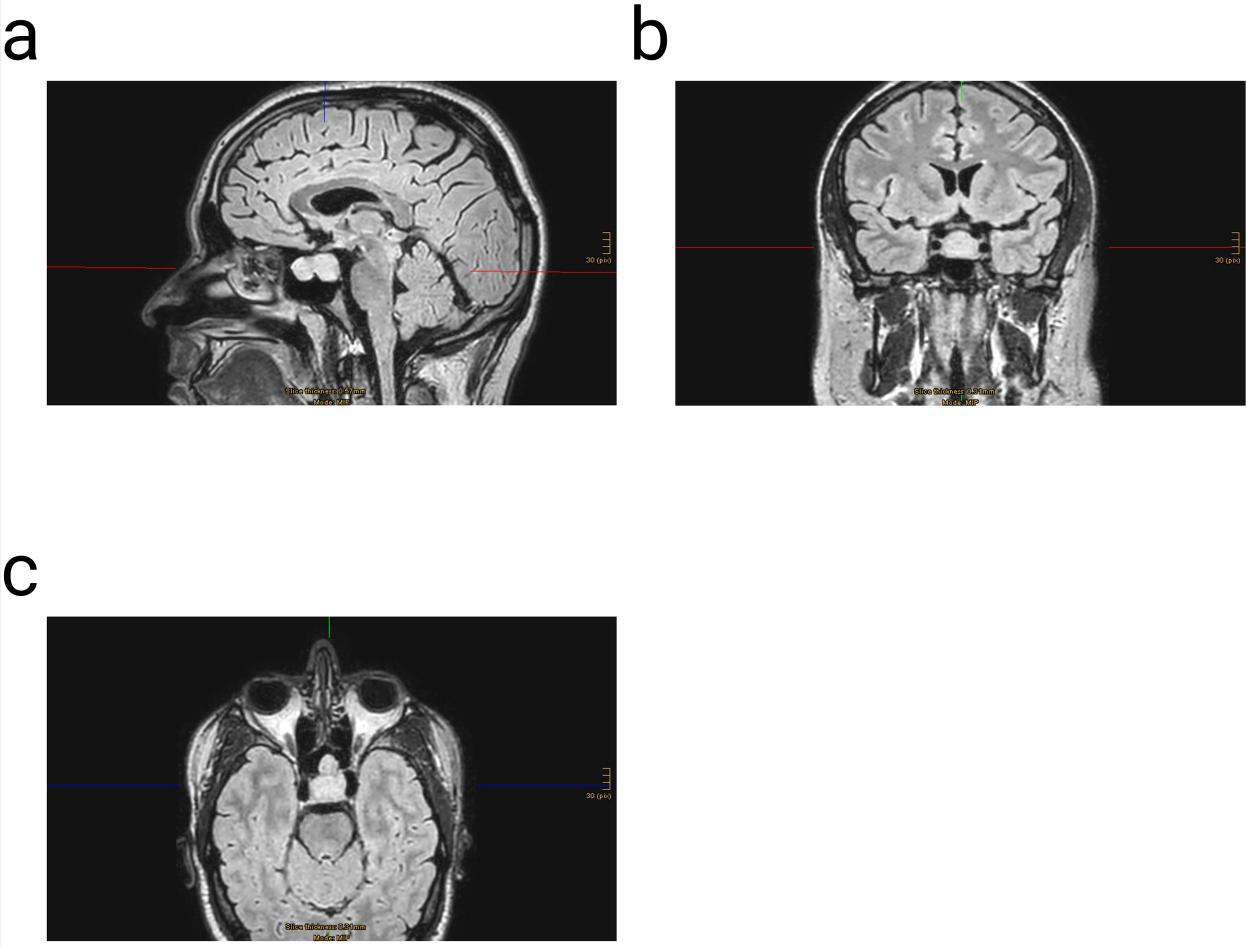
Example of a three dimensional (3D) fluid attenuated inversion recovery (FLAIR) magnetic resonance imaging (MRI) with contrast enhancement before surgery; **(a)** sagittal- **(b)** coronar- & **(c)** axial view.

**Figure 4:**
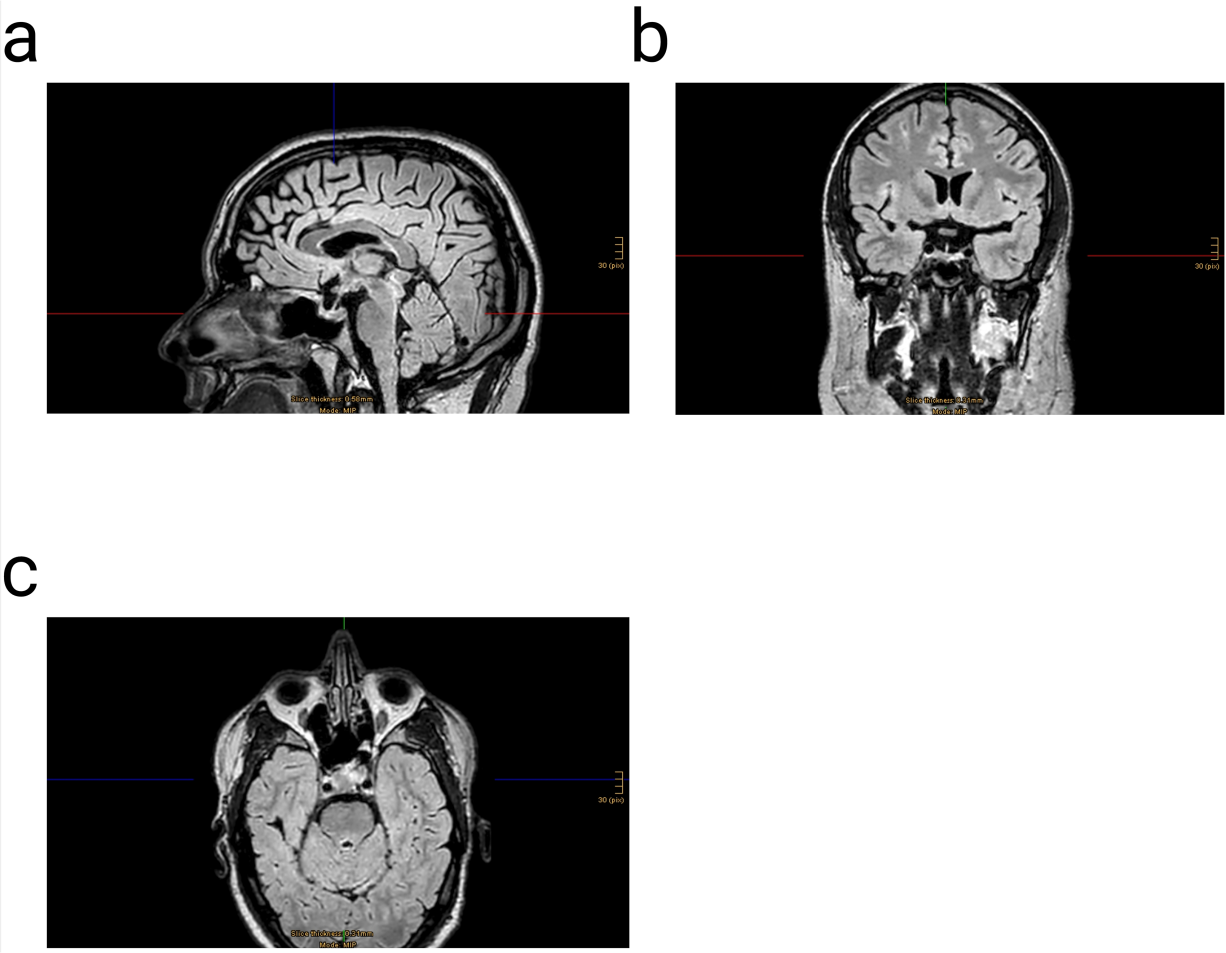
Three dimensional (3D) fluid attenuated inversion recovery (FLAIR) magnetic resonance imaging (MRI) with contrast enhancement one year after surgery of the same patient presented on Figure 3; **(a)** sagittal- **(b)** coronar- & **(c)** axial view.

The mean age was 55.1 years (±13.2). In all 22 cases, the application of the proposed three-layer PRF sellar floor reconstruction was safe and feasible. During the 12-month follow-up period, no side effects or complications associated with the PRF were observed. The intraoperative preparations of the s-PRF and i-PRF elements were conducted during surgery, and their application did not affect the duration of the procedure. None of the patients exhibited new neurological deficits in the postoperative phase. Intraoperatively, 77.3% of the patients demonstrated a cerebrospinal fluid (CSF) leak. Among these, 4.5% presented with an Esposito Grade 1, 63.6% with Grade 2, and 9% with Grade 3. One patient with an intraoperative CSF leak and Esposito Grade 3 developed a CSF leak on the first postoperative day, which was successfully managed with the placement of a lumbar drain (200 ml/24 h) for 5 days. The proposed three-layer platelet-rich fibrin (PRF) reconstructive strategy effectively prevented CSF leaks in 95% of the patients and in 94% of the subgroup with intraoperatively evident CSF leaks (Esposito Grade 1–3). The one-year follow-up magnetic resonance imaging scans (MRI) revealed no recurrence and no pathological signals in the reconstructed sellar floor (Figures 3, 4).

## Discussion

Sellar floor reconstruction is a critical component of endonasal endoscopic transsphenoidal surgery, essential for preventing complications associated with postoperative cerebrospinal fluid (CSF) leaks.

Our proposed three-layer sealing approach utilized autologous platelet-rich fibrin in solid and semifluid form to reconstruct the sellar floor. This method employed an s-PRF membrane as an inlay, a semifluid autologous bioglue intermediate layer with i-PRF, and finally another s-PRF membrane as an onlay. This strategy effectively prevented postoperative CSF leaks in 95% of our patients and in 94% of those with evident intraoperative CSF leaks (Esposito Grade 1–3). Notably, only one patient with Esposito Grade 3 (high flow) presented postoperatively with a CSF leak, which was successfully treated with a lumbar drain. No adverse effects or other postoperative complications were observed over a follow-up period of one year associated with the PRF reconstruction.

Autologous platelet-rich fibrin comprises a three-dimensional polymerized fibrin network encapsulating platelet-derived growth factors, chemokines, and leukocytes. It has demonstrated efficacy in promoting wound healing across various human tissues, and its effectiveness has been substantiated in multiple medical disciplines, including plastic, maxillofacial, orthopedic, and neurological surgery (15, 18, 30–36). Research conducted by Fredes et al. in 2017 indicated that PRF facilitated the bone healing process of the skull base following EET surgery (37), while a study by Constanzo et al. in 2022 demonstrated that the application of a single PRF membrane for sellar floor reconstruction significantly reduced the incidence of postoperative CSF leaks and exhibited no adverse effects on patients in whom a CSF leak was observed intraoperatively (11).

The proposed three-layer approach incorporating PRF during EET surgery was designed to position the inlay membrane within the border of the sellar floor opening, thereby minimizing the risk of displacement under CSF pressure and further enhancing the sealing effect with an additional layer of semi-fluid PRF-glue and another layer of PRF membrane to promote healing in the fenestrated sphenoidal sinus. Furthermore, PRF encapsulates the patient's own leukocytes within its matrix, which potentiates a local antimicrobial effect as demonstrated by Castro et al. (38). Finally, as demonstrated by Ockerman et al. in 2020, s-PRF exhibits excellent biomechanical properties, rendering it suitable for the sellar floor reconstruction (39).

A diverse array of well-established skull base reconstruction techniques following EET surgery has been proposed and evaluated over the years; however, a widely accepted reconstruction algorithm remains elusive. Hada et al. (2016) significantly advanced the field by introducing the pedicled nasoseptal flap (NSF), thereby enabling a more extensive application of this technique (40). A comprehensive multi-center study conducted by Ali et al. in 2024 employed NSF alone or in combination with other in-/on-lays for sellar floor reconstruction in 175 patients who underwent EET for the resection of hypophyseal macroadenomas. Postoperatively, only two patients developed a CSF leak but 11 developed intranasal synechia, 8 sinusitis and 19 nasal obstructions. The primary limitation of NSF is the infrequent occurrence (<1%) of necrosis resulting from compromise of the vascularized pedicle, and the fact that removal of the mucosa from the nasal septum creates a substantial exposed defect that heals through secondary intention over an extended period (41–43). In 2013, Garcia-Navarro and colleagues introduced the Gasket Seal Technique for addressing intracranial dead space (44). This method involves placing an autologous fat graft, followed by an autologous fascia lata graft that extends 1 cm beyond the bony skull base defect's circumference. An autologous bone graft or synthetic polyethylene implant is then positioned over the fascial graft, fitting precisely within the bony defect. In later cases, the authors applied a NSF over this solid support, secured with DuraSeal (Confluent Surgical, United States). The technique was often complemented by 24–48 h of prophylactic lumbar drainage, used in 67% of cases. When the gasket seal technique was combined with a NSF, the authors observed a post-operative CSF leak rate of 4.7% (44). In 2019, Cavallo et al. introduced the 3F technique for skull base reconstruction (45). The first F, representing “fat,” involves inserting an autologous fat graft into the space left after tumor removal. This graft covers the entire osteodural defect and is secured with fibrin glue. The second F, standing for “flap,” refers to the placement of the NSF, which is reinforced with cellulose sponges and held in place by nasal tamponades for 72 h. The third F, “flash,” emphasizes the importance of early patient mobilization. Patients are encouraged to sit up, walk, and maintain an upright position as soon as possible after surgery. By implementing this reconstruction protocol, the authors reported a 4% post-operative CSF leak rate in 25 patients with large osteodural defects following EET (45). Notably, post-operative lumbar drainage was not used in this approach. In summary the aforementioned techniques have shown efficiency in reducing the occurrence of postoperative CSF leaks after EET surgery, but significantly increase the duration of surgery and require harvesting of fat or fascia combined with other synthetic biomaterials. Furthermore, Di Giorgio et al. recently demonstrated that EET in conjunction with various reconstructive strategies, such as NSF and the Gasket-Seal technique, constitutes a safe and effective treatment modality even for pediatric middle skull base pathologies, yielding high success rates with associated complications of less than 3% (46).

Our proposed method is fully orthobiological, following the current strategy of reconstructing the sellar floor in layers. It supplies numerous autologous growth factors locally, potentially improving healing, and autologous leukocytes offer infection protection (23).

The primary limitation of this investigation is the absence of matched controls and the small sample size. Another constraint is that this technique was applied exclusively to macroadenomas of the pituitary gland and not to other extensive pathologies of the pituitary gland that necessitate a broader surgical corridor and are more frequently associated with high-flow intraoperative cerebrospinal fluid leaks. A significant limitation of this study is the exclusion of patients undergoing anticoagulant therapy. As demonstrated by Ockerman et al. in 2020, anticoagulant treatments may influence the mechanical stability of s-PRF and the polymerization time of i-PRF. Therefore, future research should address this limitation and determine which patient populations are suitable candidates for this technique. Furthermore, our institutional standards, in addition to a follow-up MRI scan, do not include any CT scan follow-ups, thus precluding an evaluation of the bone regeneration of the sellar floor. As demonstrated by Gerardi et al. in a systematic review from 2023, the combination of deproteinized bovine bone materials (DBBM) with PRF presented a viable alternative in the bone regeneration procedure for maxillary sinus floor lift techniques and demonstrated efficacy in enhancing the healing process and mitigating intraoperative complications such as orosinus perforations or postoperative complications (47).

Based on this preliminary experience with PRF, we aim to conduct subsequent comparisons with other established reconstructive strategies and validate its efficacy through randomized controlled trials.

## Conclusion

A three-layered autologous platelet rich fibrin reconstructive strategy for the sellar floor following endonasal endoscopic transsphenoidal surgery represents a promising, novel, and safe approach that may reduce the incidence of postoperative cerebrospinal fluid leaks.

## Data Availability

The original contributions presented in the study are included in the article/Supplementary Material, further inquiries can be directed to the corresponding author.
